# Drug Combination Studies of Isoquinolinone AM12 with Curcumin or Quercetin: A New Combination Strategy to Synergistically Inhibit 20S Proteasome

**DOI:** 10.3390/ijms251910708

**Published:** 2024-10-04

**Authors:** Carla Di Chio, Santo Previti, Josè Starvaggi, Fabiola De Luca, Maria Luisa Calabrò, Maria Zappalà, Roberta Ettari

**Affiliations:** Department of Chemical, Biological, Pharmaceutical, and Environmental Sciences, University of Messina, Viale Ferdinando Stagno d’Alcontres 31, 98166 Messina, Italy; carla.dichio@unime.it (C.D.C.); spreviti@unime.it (S.P.); starvaggi4@gmail.com (J.S.); fabiola.deluca@studenti.unime.it (F.D.L.); mlcalabro@unime.it (M.L.C.); rettari@unime.it (R.E.)

**Keywords:** 20S proteasome, chymotrypsin-like activity, curcumin, quercetin, combination studies

## Abstract

In the eukaryotic cells, the ubiquitin–proteasome system (UPS) plays a crucial role in the intracellular protein turnover. It is involved in several cellular functions such as the control of the regular cell cycle progression, the immune surveillance, and the homeostasis. Within the 20S proteasome barrel-like structure, the catalytic subunits, β1, β2 and β5, are responsible for different proteolytic activities: caspase-like (C-L), trypsin-like (T-L) and chymotrypsin-like (ChT-L), respectively. The β5 subunit is particularly targeted for its role in antitumor activity: the synthesis of β5 subunit inhibitors could be a promising strategy for the treatment of solid and hematologic tumors. In the present work, we performed two combination studies of **AM12**, a recently developed synthetic proteasome inhibitor, with curcumin and quercetin, two nutraceuticals endowed of many pharmacological properties. We measured the combination index (CI), applying the Chou and Talalay method, comparing the two studies, from 50% to 90% of proteasome inhibition. In the case of the combination **AM12** + curcumin, an increasing synergism was observed from 50% to 90% of proteasome inhibition, while in the case of the combination **AM12** + quercetin an additive effect was observed only from 50% to 70% of β5 subunit inhibition. These results suggest that combining **AM12** with curcumin is a more promising strategy than combining it with quercetin for potential therapeutic applications, especially in treating tumors.

## 1. Introduction

The ubiquitin–proteasome system (UPS) is indeed a vital component of eukaryotic cells, serving as the primary non-lysosomal proteolytic pathway responsible for degrading unwanted or misfolded proteins [1,2]. It plays key roles in regulating various cellular processes such as cell cycle progression, immune responses, and maintaining cellular homeostasis [3,4]. Structurally, the 26S proteasome is composed of a 20S catalytic core and two 19S regulatory caps [5,6]. The 20S core has a typical barrel-like structure, consisting of four overlapping rings: two outer rings made up of seven α-subunits and two inner rings composed of seven β-subunits [7]. The α-subunits are mainly structural, helping to form the overall barrel shape and control access to the proteolytic chamber. The β-subunits are responsible for the proteolytic functions of the proteasome, with the β1 subunit exhibiting caspase-like (C-L) activity, the β2 subunit showing trypsin-like (T-L) activity, and the β5 subunit displaying chymotrypsin-like (ChT-L) activity [8]. Each of the three proteasome catalytic subunits (β1, β2 and β5) possesses a catalytic site that uses the nucleophilic γ-hydroxyl group of the *N*-terminal threonine (Thr) to break peptide bonds. This mechanism is a key feature of the proteasome proteolytic activity, enabling the degradation of damaged or misfolded proteins within the cell [3,4].

Dysfunctions in the UPS can indeed contribute to the onset of tumors, particularly in the field of hematological malignancies [6,9,10]. The UPS is essential for maintaining cellular homeostasis by regulating protein degradation. When this system is impaired, it can result in the accumulation of damaged or misfolded proteins, leading to cellular stress and potentially contributing to oncogenesis.

Several studies have shown that selective inhibition of the β5 subunit, which is responsible for the ChT-L activity, can induce cytotoxic effects specifically in tumor cells [11]. This selective targeting is beneficial because it can kill cancer cells while sparing healthy cells. In contrast, inhibiting all three catalytic subunits of the proteasome (β1, β2, and β5) can result in a non-selective cytotoxic effect, affecting both cancerous and healthy cells. This is why therapies aimed at selectively inhibiting the β5 subunit are considered promising for cancer treatment, especially in hematologic malignancies [11].

Bortezomib (BZB) became the first proteasome inhibitor approved in 2003 for the treatment of multiple myeloma and mantle cell lymphoma [12]. Several ongoing studies are exploring Bortezomib’s potential for treating solid tumors [13,14]. Recent research has particularly shown its efficacy in hepatocellular carcinoma (HCC) [15]. In preclinical and early clinical trials, Bortezomib has demonstrated promising antitumor effects both as a standalone treatment and in combination with other therapeutic agents [16,17].

**Figure 1:**
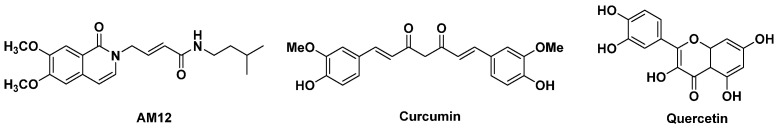
Chemical structures of **AM12**, curcumin and quercetin.

Our research group has been actively working on developing covalent and non-covalent proteasome inhibitors [18,19,20,21,22,23,24], and one of the recent compounds, **AM12**, was found to be particularly promising (Figure 1) [18]. Indeed, **AM12** showed a *K*_i_ value of 1.18 µM towards the β5 subunit, along with no inhibition and a lower binding affinity towards β1 and β2 subunits, respectively.

In recent years, the combination of synthetic drugs with nutraceuticals has gained significant attention for the treatment of several diseases [25,26,27,28,29,30]. The simultaneous employment of two or more drugs has become a key strategy in several areas, such as cancer, metabolic disorders, and infectious diseases, among others [31]. Relevant advantages, such as the increased efficacy, reduced drug resistance, lower doses of individual drugs, broader spectrum of action, overcoming limitations of monotherapy, reduced metabolic rate, and delayed disease progression, make the use of bioactive molecules in combination a valuable strategy in the management of the whole health system [32].

Given our research group’s expertise in drug combination studies [33,34,35,36], we have conducted two combination studies involving the synthetic proteasome inhibitor **AM12** with two well-known nutraceuticals, namely curcumin and quercetin (Figure 1).

In this study, we utilized the Chou–Talalay method, a widely recognized approach for evaluating drug interactions, to examine the effects of combining **AM12** with either curcumin or quercetin. This method determines the combination index (CI), which reveals the nature of the interaction between the compounds: a CI value of < 1 indicates synergism, = 1 suggests an additive effect and > 1 signifies antagonism [37,38].

The findings from this analysis enabled a detailed comparison of the two combinations, **AM12** + curcumin and **AM12** + quercetin, to determine which exhibited a more favorable effect. The results of this investigation are herein reported.

## 2. Results

**Figure 2:**
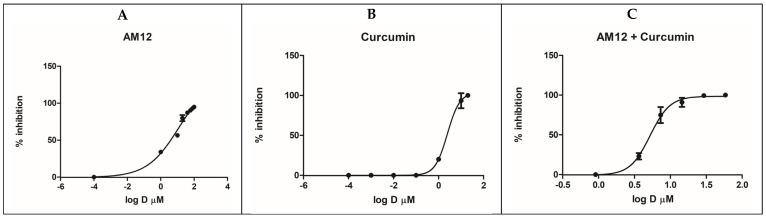
Dose–response curves for β5 inhibition by **AM12** (**A**), curcumin (**B**) and **AM12** + curcumin in combination (**C**). Each experiment was performed two times, each in duplicate.

**AM12** and curcumin were tested on the recombinant human erythrocyte proteasome β5 subunit using Suc-Leu-Leu-Val-Tyr-AMC as a fluorogenic substrate. The experiments involved both individual testing and combination studies to evaluate the inhibitory effects and potential synergism of **AM12** and curcumin. Initially, a preliminary screening was carried out at concentrations of 100 µM, 1 µM and 0.1 µM, to define the range of activity of the two inhibitors. **AM12** and curcumin were then tested separately, in two independent experiments, each of which was performed in duplicate. These experiments were carried out by selecting seven different concentrations, ranging from the minimum dose inhibiting the enzyme up to the maximum dose, which completely inhibits the activity of the proteasome. **AM12** was tested in the range 100–1 µM, while curcumin was tested in the range 20–0.0001 µM. The IC_50_ values were then calculated from the dose–response curves, as reported in Figure 2A,B, obtaining values IC_50_ of 12.17 ± 1.80 µM for **AM12** and 2.46 ± 0.93 µM for curcumin. These findings indicate that curcumin is approximately five-times more potent than **AM12** in inhibiting the β5 subunit under the conditions tested. Subsequently, six concentrations were chosen for the compound combinations (1/16 × IC_50_, 1/4 × IC_50_, 1/2 × IC_50_, IC_50_, 2 × IC_50_, and 4 × IC_50_, Table 1), to evaluate whether a synergistic, additive, or antagonistic effect in the combination study of the two inhibitors against the β5 subunit of the human proteasome could occur.

**Figure 3:**
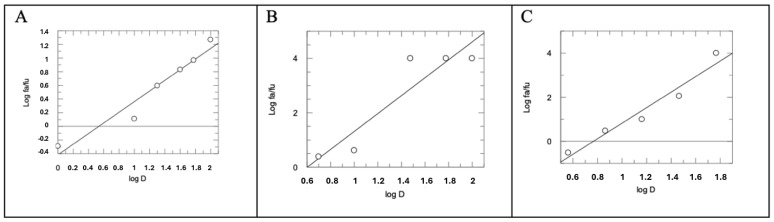
Median Effect Plot for **AM12** (**A**), curcumin (**B**) and **AM12** + curcumin in combination (molar ratio 4.95:1) (**C**). D is the dose, and fa and fu the affected and the unaffected fraction of β5 activity, respectively, by the dose D.

In this assay, the combination of **AM12** + curcumin (molar ratio 4.95:1) provided an IC_50_ value of 5.21 ± 0.45 µM (Figure 2C). Subsequently, each dose–response curve (Figure 2A–C) was converted into the Median Effect Plot, obtained by plotting the log (fa/fu) on the y-axis versus the log (D) on the x-axis (Figure 3A–C).

In this plot the maximum response corresponds to 1, unlike the dose–response curve where it corresponds to 100. Therefore, fa + fu = 1, where fa corresponds to the “affected fraction” of enzyme, while fu is the “unaffected fraction”, i.e., the residual enzymatic activity. The slope of the straight line of each Median Effect Plot corresponds to the “m” value, when m is the Hill-type coefficient signifying the sigmoidicity of the dose–effect curve. In detail, **AM12** showed a value of m_1_ = 0.7785, curcumin a value of m_2_ = 3.2946, while the combination assay showed a value of m_1,2_ = 3.5279, with an **AM12**/curcumin molar ratio of 4.95:1.

Once the three different m values were calculated by Grafit software, we established the doses capable of inducing each percentage of proteasome inhibition, using the Median Effect Equation: D = IC_50_ [fa/fu]1/m [37,38].

In the first part of our study, comparing the IC_50_ of **AM12**, curcumin, and the combination of **AM12** + curcumin (Figure 2) and the related m values to each Median Effect Plot (Figure 3), the following was found: for **AM12,** an IC_50_ = 12.17 ± 1.80 µM and m_1_ = 0.7785, for curcumin, an IC_50_ = 2.46 ± 0.93 µM and m_2_ = 3.2946 and for the combination of **AM12** + curcumin (molar ratio 4.95:1), an IC_50_ = 5.215 ± 0.45 µM and m_1,2_ = 3.5279.

**AM12** and quercetin were also tested on the recombinant human erythrocyte proteasome β5 subunit using Suc-Leu-Leu-Val-Tyr-AMC as a fluorogenic substrate. Initially, a preliminary screening was carried out at concentrations of 100 µM, 1 µM, and 0.1 µM, to define the range of activity of quercetin.

Quercetin was tested separately in two independent experiments, each of which was performed in duplicate. In the above experiments, seven concentrations were selected for testing quercetin. In particular, quercetin was tested in the range 100–0.001 µM, while experiments with **AM12** alone are already described above.

**Figure 4:**
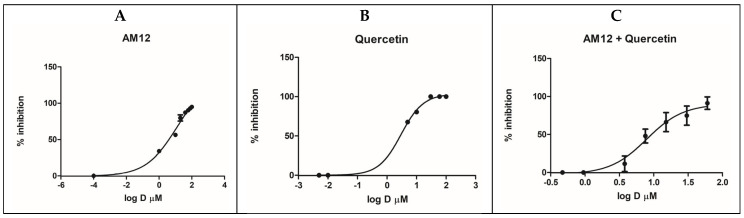
Dose–response curves for β5 inhibition by **AM12** (**A**), quercetin (**B**) and **AM12** + quercetin in combination (**C**). Each experiment was performed two times, each in duplicate.

The respective IC_50_ values were calculated from the dose–response curves (Figure 4A–C). For quercetin, with these new measurements an IC_50_ value of 2.96 ± 0.77 µM was obtained, while for **AM12** the IC_50_ was equal to 12.17 ± 1.80 µM, as described above. In a subsequent experiment, six concentrations were established for the combination **AM12** + quercetin (1/16 × IC_50_, 1/4 × IC_50_, 1/2 × IC_50_, IC_50_, 2 × IC_50_, and 4 × IC_50_, Table 2), with the aim to evaluate whether a synergistic, an additive, or an antagonistic effect occurs in the combination study between the two inhibitors of the β5 subunit of the human erythrocyte proteasome.

In this assay, the combined doses of **AM12** + quercetin (molar ratio 4.11:1) provided an IC_50_ value of 7.89 ± 2.52 µM. (Figure 4C).

**Figure 5:**
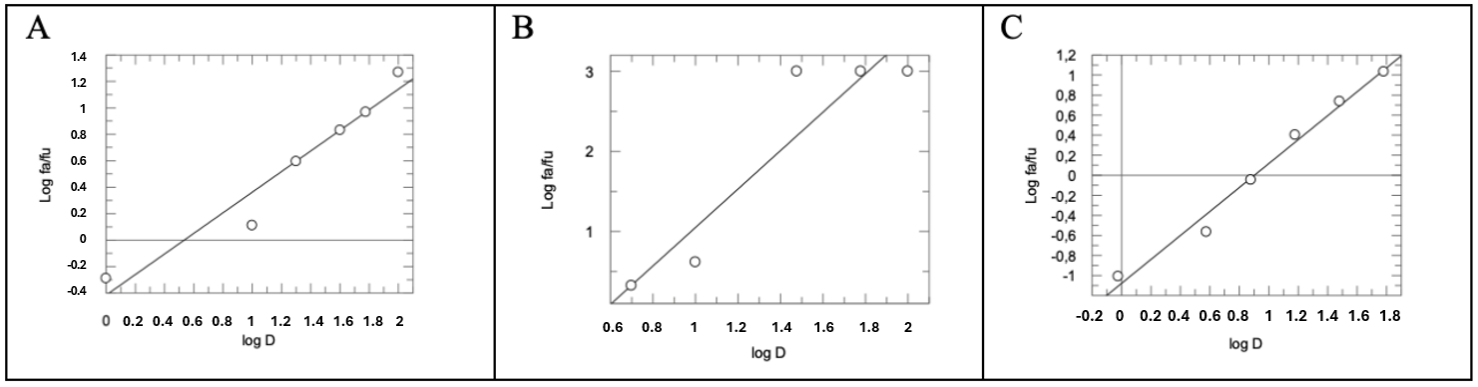
Median Effect Plot for **AM12** (**A**), quercetin (**B**) and **AM12** + quercetin in combination (molar ratio 4.95:1) (**C**). D is the dose, and fa and fu the affected and the unaffected fraction of β5 activity, respectively, by the dose D.

Subsequently, also in the second study each dose–response curve (Figure 4A–C) was converted into the Median Effect Plot (Figure 5A–C), thus obtaining for **AM12** a value of m_1_ = 0.7785, for quercetin a value of m_2_ = 2.4004, while for the combination assay it was found that m_1,2_ =1.2958, with an **AM12**/quercetin molar ratio of 4.11:1.

By comparing the IC_50_ (Figure 4A–C) of **AM12**, quercetin, and the combination of **AM12** + quercetin and the relative m values obtained from each Median Effect Plot (Figure 5A–C), an IC_50_ = 12.17 ± 1.80 µM and m_1_ = 0.7785 were found for **AM12**, an IC_50_ = 2.96 ± 0.77 µM and m_2_ = 2.4004 for quercetin, and an IC_50_ = 7.899 ± 1.80 µM and m_1,2_ = 1.2958 for the combination of **AM12** + quercetin (molar ratio 4.11:1).

**Figure 6:**
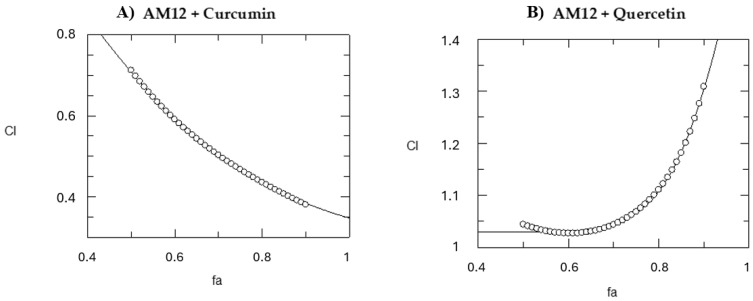
Computer-generated graphical presentation of the combination index (CI) vs. the fraction affected (fa), i.e., the effect of reduction in proteasome activity exerted by **AM12** + curcumin (**A**) and by **AM12** + quercetin (**B**). The circles indicate the trend of the combination index.

Then, we calculated the CI, which expresses the nature of the inhibition towards the target enzyme when the two drugs are tested in combination. It is well established that CI > 1 indicates an antagonistic effect, CI = 1 represents an additive effect, whereas CI < 1 suggests a synergistic effect [37,38]. The CI for mutually exclusive drugs, which act independently, was calculated. This method provides a quantitative assessment of how two compounds properly work together. To determine the CI, we used the Grafit software, and the CI was calculated from 50% to 90% of the inhibition of the β5 subunit of the human proteasome for both the combinations **AM12** + curcumin and **AM12** + quercetin (Table 3 and Figure 6A). In the case of the combination **AM12** + curcumin from 50% to 90%, an increasing synergistic action was observed, while in the case of the combination **AM12** + quercetin from 50% to 70% only an additive effect was observed (Table 3 and Figure 6B), which then converted into a slight antagonism from 80% to 90% of proteasome inhibition.

## 3. Discussion

In this study, we tested the synthetic proteasome inhibitor **AM12** in combination with two nutraceuticals: curcumin and quercetin.

**Scheme 1:**
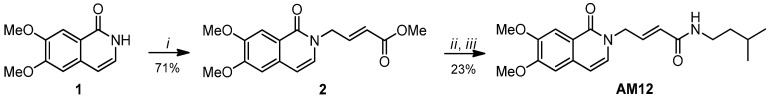
Reagents and conditions: (*i*) NaH, N_2_, dry DMF, 0 °C, **1**, 1 h, and then methyl 4-bromocrotonate, 0 °C to room temperature (rt), 12 h; (*ii*) MeOH, LiOH, 0° C to rt, TLC monitoring; and (*iii*) dry DMF, resulting acid from **2**, HATU, 0 °C, 10 min and then DIPEA, iso-pentyl amine, 0 °C to rt, 12 h.

Structurally, **AM12** is an isoquinolinone derivative with a bicyclic structure featuring two methoxy groups and an allylic spacer, which connects the heterocycle to an amide function with an aliphatic substituent, i.e., the isopentyl, preferred by the β5 catalytic subunit of proteasome. **AM12** was obtained using a synthetic pathway of three steps, and only two chromatography purifications were required: the overall yield resulted to be 16.3% (Scheme 1).

Despite the presence of a Michael acceptor moiety, i.e., the α,β-unsaturated amide, the obtained experimental data suggest a non-time dependent, reversible inhibition of the enzymatic activity of proteasome. This reversibility could offer advantages in therapeutics, such as better control of overdosing, reduced risk of off-target effects due to covalent modification of unintended proteins and the potential for fine-tuning inhibitor selectivity by optimizing the reversible interaction. Molecular modeling studies highlighted that **AM12** positions its Michael acceptor group far from the catalytic Thr1, ruling out the possibility of this compound to act as a covalent inhibitor. In the β5 subunit, the binding of **AM12** is stabilized by interactions of the isopentyl group at the P3 position with residues Ala20, Ala22, Ala27 and Val31. Additionally, two water-mediated hydrogen bonds are established: one between the carbonyl oxygen of the amide bond and the side chain of Lys136, and another between the isoquinolone lactam oxygen and Asp125. The isoquinolone core also engages in hydrophobic contacts with Val127 and Pro126. The presence of the isopentyl group was found to be crucial for the β5 subunit inhibition. Indeed, the incorporation of linear and cyclic aliphatic residues, as well as the introduction of phenyl-containing substituents, led to a significant loss of affinity [18]. Additionally, **AM12** showed no biding affinities against the β1 and β2 subunits. In this case, the 6,7-dimethoxy-1-oxoisoquinolinone core seems to be the main responsible for the selectivity towards the β5 subunit, since **AM12** analogues, which differ for the *N*-anchored substituents, exhibited no inhibitory activity or a low percentage of inhibition towards β1 and β2 subunits.

Curcumin, a bioactive compound derived from the spice turmeric (*Curcuma longa*), is well known for its strong anti-inflammatory, antioxidant, antitumor and antimicrobial properties [39,40]. Curcumin has been extensively biologically characterized for its potential therapeutic benefits in a range of conditions, such as cancer, neurodegenerative diseases, cardiovascular disorders, and inflammatory diseases [41,42]. Curcumin exerts its effects through multiple mechanisms, such as inhibiting pro-inflammatory cytokines, modulating oxidative stress, and interfering with cancer cell proliferation and survival pathways, such as NF-κB and PI3K/Akt [40]. Some studies suggest that curcumin may influence proteasome activity indirectly by affecting proteasome assembly or through interactions with regulatory components rather than by direct inhibition of specific subunits like β5 [43,44,45]. As such, while curcumin’s role in proteasome inhibition might not be extensively characterized in terms of specific subunit inhibition, its overall impact on proteasome function remains a point of interest for its potential therapeutic benefits. Despite its broad pharmacological potential, curcumin’s poor bioavailability is a limitation, leading to efforts to enhance its absorption through formulations or combinations with other agents [46,47]. In combination with other drugs or compounds, such as proteasome inhibitors like **AM12**, curcumin may enhance therapeutic efficacy or reduce the required dose of the drug, potentially minimizing side effects.

Quercetin is one of the most abundant flavonoids, found in a variety of fruits, vegetables and grains, such as apples, onions, and berries. It is renowned for its antioxidant, anti-inflammatory, and antimicrobial properties [48]. Quercetin has been studied for its potential benefits in numerous health conditions, including cardiovascular diseases, cancer, and allergies [49]. Quercetin has demonstrated potential in preclinical studies for its positive effects to promote apoptosis in cancer cells, inhibit tumor growth, and suppress metastasis [50,51]. Several studies have reported that the quercetin effects on apoptosis are mediated by the inhibition of the proteolytic activity of proteasome [52,53]. However, the exact mechanism by which quercetin inhibits proteasomal activity has not been fully elucidated. Quercetin’s proteasome-inhibitory effects can potentially enhance the efficacy of other therapeutic agents, such as proteasome inhibitors like **AM12**, where it may help enhance therapeutic outcomes and improve cancer treatment efficacy.

The aim of this study was to assess the potential for synergy, additive effects, or antagonism when **AM12** was separately combined with curcumin or quercetin.

In the case of the combination of **AM12** with curcumin, a progressive enhancement in synergistic activity was observed as the level of proteasome inhibition increased from 50% to 90% (Table 3). Indeed, when the fa was equal to 0.5, the interaction between **AM12** and curcumin demonstrated moderate synergy, which progressively intensified as the inhibition level approached higher fa values (0.9). This suggests that the combination of these two agents becomes increasingly effective at higher doses, potentially amplifying their therapeutic impact on proteasome activity. This synergistic interaction may be indicative of complementary mechanisms of action, with curcumin enhancing the efficacy of **AM12** in a dose-dependent manner. **AM12**, when tested individually, exhibited an m_1_ value equal to 0.7785. This indicates a negative cooperativity in its dose–response curve, meaning that as **AM12** binds to the β5 subunit, it may reduce the likelihood of a hypothetical subsequent binding. The dose–response curve for **AM12** is not highly sigmoidal (Figure 2A and Figure 4A) but rather shows a more gradual slope, suggesting that the compound’s binding or inhibitory action may not follow a strongly cooperative mechanism. On the other hand, curcumin showed a significantly higher m_2_ value of 3.2946. These data suggest a strong positive cooperativity, where the binding of one curcumin molecule enhances the likelihood of additional curcumin molecules binding to the β5 subunit. This steep dose–response curve suggests that curcumin’s inhibitory effects become much more pronounced after reaching a certain threshold concentration, likely leading to a sharp increase in proteasome inhibition once multiple binding sites are occupied. For the combination assay of **AM12** and curcumin, the m_1,2_ value was determined to be 3.5279, with a molar ratio of 4.95:1 (**AM12**:curcumin). This m value, which is even higher than curcumin’s individual m_2_, suggests that the combination of the two inhibitors induces a more cooperative and steeply sigmoidal dose–response curve than either compound alone. The elevated Hill coefficient indicates a strong synergistic interaction between **AM12** and curcumin in combination, where the binding of one compound enhances the inhibitory effect of the other, leading to an amplified response. The molar ratio of 4.95:1 reflects the proportional amounts of **AM12** and curcumin used in the combination assay, and it could be optimized based on the observed cooperativity to maximize the synergistic effect. This combination may potentially result in more effective proteasome inhibition at lower concentrations compared to either compound used individually, which is an important factor when considering dose optimization and minimizing toxicity in therapeutic applications.

In contrast, the combination of **AM12** with quercetin exhibited a different interaction profile. At proteasome inhibition levels of 50% to 70%, the interaction between **AM12** and quercetin was predominantly additive. However, as the inhibition levels reached 80% to 90%, the interaction shifted towards slight antagonism, suggesting that higher concentrations of quercetin may interfere with or diminish the efficacy of **AM12** at these concentrations. This shift from an additive to antagonistic effect could be due to complex biological interactions, possibly involving competitive or overlapping pathways between quercetin and **AM12** at higher proteasome inhibition thresholds. By analyzing the IC_50_ values and m values from Figure 4 and Figure 5, respectively, important conclusions about the potency and dose–response characteristics of **AM12** and quercetin alone and their combination could be drawn. As above-mentioned, **AM12** showed an IC_50_ value of 12.17 ± 1.80 µM and an m_1_ equal to 0.7785, which indicates a relatively higher concentration to inhibit 50% of proteasome activity and a negative influence for the likelihood of subsequent binding. Quercetin showed an IC_50_ of 2.96 ± 0.77 µM and an m_2_ value of 2.4004. Therefore, quercetin is more potent than **AM12** for the inhibition of the β5 subunit of proteasome. The m_2_ value of 2.4004 is notably higher than 1, suggesting a strong positive cooperativity. These data indicate that quercetin exhibits a steep, sigmoidal dose–response curve, where the binding of one quercetin molecule enhances further binding, resulting in a sharp increase in inhibitory activity once a certain threshold concentration is reached. The combination of **AM12** + quercetin yielded an IC_50_ of 7.9 ± 1.80 µM and an m_1,2_ value of 1.2958 (molar ratio of 4.11:1). The IC_50_ of the combination is lower than that of **AM12** alone but higher with respect to that of quercetin alone. However, the m_1,2_ value of 1.2958 indicates a more cooperative, but not extremely sigmoidal, interaction in the combination. Overall, quercetin alone shows a greater potency and stronger positive cooperativity than **AM12**, while the combination of **AM12** and quercetin improves the potency of **AM12** but does not fully match the strong positive cooperativity exhibited by quercetin on its own. The combination presents a moderately cooperative dose–response profile, which could offer a balanced approach to achieving effective proteasome inhibition with potentially lower toxicity.

## 4. Materials and Methods

**AM12** was synthesized by our research group, as we already reported [18]. Synthesis was carried out starting from the commercially available starting material (i.e., 6,7-dimethoxyisoquinolin-1(2*H*)-one, Sigma Aldrich-Merck Life Science, Milan, Italy) in a scale of 5 mmol and 288 mg were obtained. NMR spectra, yields, retention factors, and consistency were comparable with those known in the literature. The overall yield was confirmed (17.1% vs 16.3%). Curcumin and quercetin were purchased from Sigma Aldrich-Merck Life Science (Milan, Italy). The human erythrocyte 20S proteasome was purchased from Enzo Life Science (Farmingdale, NY, USA).

Preliminary screening on the 20S proteasome was performed at a concentration of 100 µM, 1 µM and 0.1 µM to identify the range of activity of **AM12**, curcumin and quercetin. An equivalent amount of DMSO (Sigma Aldrich-Merck Life Science, Milan, Italy) was used as a negative control; meanwhile, the proteasome inhibitor **MG-132** (Cbz-Leu-Leu-Leu-H, Sigma Aldrich-Merck Life Science, Milan, Italy) was used as the positive control. The product release from hydrolysis of the fluorogenic substrate specific for the chymotrypsin-like (ChT-L) activity of the constitutive proteasome (Suc-LLVY-AMC, Bachem, Bubendorf, Switzerland) was determined continuously over a period of 10 min at 30 °C. The assay buffer is composed of 50 mM Tris-HCl, pH 7.5, 0.03% SDS (Sigma Aldrich-Merck Life Science, Milan, Italy). Stock solutions for the three inhibitors were prepared at 20 mM in DMSO. In particular, 2.1 mg of **AM12**, 1.5 mg of curcumin, and 1.9 mg of quercetin were diluted in 292.94, 203.59, and 314.32 µL of DMSO, respectively. Starting from these, the appropriate dilutions led to the desired concentrations.

In the various assays, **AM12**, curcumin, and quercetin were tested separately twice in duplicate in 96-well plates (BRAND^®^, Wertheim, Germany) in a total volume of 200 µL. The following concentrations were used: (*i*) for **AM12,** 100 µM, 80 µM, 60 µM, 40 µM, 20 µM, 10 µM, and 1 µM; (*ii*) for curcumin, 20 µM, 10 µM, 5 µM, 1 µM, 0.1 µM, 0.01 µM, and 0.001 µM; and (*iii*) for quercetin, 100 µM, 10 µM, 5 µM, 1 µM, 0.1 µM, 0.01 µM, and 0.001 µM.

The fluorescence of the product AMC (7-amino-4-methylcoumarin) released from substrate hydrolysis was measured using an Infinite 200 PRO microplate reader (Tecan, Männedorf, Switzerland). The measurements were taken at room temperature with an excitation filter set to 380 nm and an emission filter set to 460 nm, which are appropriate for detecting AMC’s fluorescent signal. Results are expressed as IC_50_ values ± SD and have been calculated by fitting the progress curves to the four-parameter IC_50_ Equation by GraphPad Prism 5.0.3 (GraphPad software Inc., San Diego, CA, USA) (1):

(1) $$y=\frac{y_{max}-y_{min}}{1+{\left(\frac{\left[I\right]}{{IC}_{50}}\right)}^{s}}+y_{min}$$

with y [∆F/min] as the substrate hydrolysis rate, y_max_ as the maximum value of the dose−response curve, measured at an inhibitor concentration of [I] = 0 µM, with y_min_ as the minimum value, obtained at high inhibitor concentrations, and s as the Hill coefficient.

The inhibitory constants (*K*_i_) were calculated according to the Cheng–Prusoff Equation (2):

*K*_i_ = IC_50_/(1 + [S]/*K*_m_) (2)

with [S] as the substrate concentration and *K*_m_ as the Michaelis–Menten constant.

For the calculation of the combination index of the combination **AM12** + curcumin or quercetin, six data points were used: 1/32 × IC_50F1+F2_, 1/4 × IC_50F1+F2_, 1/2 × IC_50F1+F2_, IC_50F1+F2_, 2 × IC_50F1+F2_, and 4 × IC_50F1+F2_, where F1 = **AM12** while F2 = curcumin or quercetin (See Table 1 and Table 2). Once the IC_50_ values ± SD for the combination were calculated, each dose–response curve was converted into the corresponding Median Effect Plot, where the maximum response is 1 instead of 100 of the dose–response curve. The fraction of the enzyme that is inhibited is named “affected fraction” (fa), while the fraction of the enzyme that is not inhibited is named “unaffected fraction” (fu), where fa + fu = 1. The Median Effect Plot is obtained by plotting the log (fa/fu) versus the log (D) on the x-axis, in such a way to calculate the “m value”, which represents the Hill-type coefficient, which means the sigmoidal trend (or S-shape) of the dose–response curve.

Once the three different m values were calculated by Grafit software (Version 5.0; Erithacus Software Limited, East Grinstead, West Sussex, UK), we established by means of the Median Effect Equation (3) the single doses that are able to inhibit the enzyme for a specific percentage of inhibition [37,38]:

D = IC_50_ [fa/fu]^1/m^ (3)

The Chou–Talalay method was then applied to evaluate multiple drug effects [37,38]. The CI for mutually exclusive drugs, which act independently, was calculated on the basis of the following Equation (4):

CI = [(D)_1_/(IC_50_)_1_] + [(D)_2_/(IC_50_)_2_] (4)

where the (IC_50_)_1_ and (IC_50_)_2_ were obtained by dose–response curves, and D_1_ and D_2_ are the concentrations able to induce a specific percentage of human erythrocyte proteasome inhibition obtained by the Median Effect Equation (3).

## 5. Conclusions

This study investigated the combination of the synthetic proteasome inhibitor **AM12** with the nutraceuticals curcumin and quercetin to evaluate their combined effects. The combination of **AM12** with curcumin showed a pronounced synergistic interaction that enhances proteasome inhibition, particularly at higher doses, indicating complementary mechanisms of action. In contrast, the combination of **AM12** with quercetin exhibited an initially additive effect that shifts to slight antagonism at higher concentrations, underscoring the complex interactions that can arise between these agents. The observed differences in dose–response profiles highlight the necessity of optimizing dosages to maximize the inhibitory properties. Ultimately, these findings suggest that the use of the selective inhibitor of the β5 site of the proteasome alongside the selected nutraceuticals can significantly influence treatment outcomes and warrant further investigation for clinical applications.

Future studies will focus on testing the cytotoxic effects of individual inhibitors and the **AM12** + curcumin combination on multiple myeloma and solid tumor cell cultures. A key goal of these studies will be to assess whether combining **AM12** with curcumin can reduce toxicity, which is a critical concern in cancer therapy. This approach aligns with the broader therapeutic strategy of using drug combinations to enhance efficacy while minimizing adverse effects, potentially leading to more effective and safer anti-tumor treatments.

Finally, it is important to highlight that, despite the current reference models for evaluating drug synergism having advanced, all of them, including the Chou and Talalay method, still face significant limitations that can impact the interpretation of obtained data about the combination [54]. Indeed, the term synergy is often misused or poorly defined, leading to misconceptions about the true nature of drug interactions. Additionally, a lack of standard reference models and the complexities involved in optimizing dose ratios hinder effective synergy analysis. Another important limitation is the need to optimize the dose ratios in drug combinations, as the interaction of two drugs can create a new dose–effect relationship, necessitating experiments to identify the most effective ratios for achieving synergy before advancing to the in vivo tests and clinical trials. Therefore, despite the progress, existing models for evaluating drug interactions still have limitations, necessitating careful selection based on the study type and future research should integrate various approaches to rigorously assess synergism and elucidate mechanisms of action, potentially utilizing in silico models for further evaluation.

## Figures and Tables

**Table 1:** Six selected doses for the combination experiments of **AM12** + curcumin.

Compound	0.062 × IC_50_	0.25 × IC_50_	0.5 × IC_50_	IC_50_	2 × IC_50_	4 × IC_50_
Curcumin	0.15 µM	0.61 µM	1.23 µM	2.46 µM	4.92 µM	9.84 µM
**AM12**	0.76 µM	3.04 µM	6.08 µM	12.17 µM	24.34 µM	48.68 µM
Curcumin + **AM12**	0.15 + 0.76 µM	0.61 + 3.04 µM	1.23 + 6.08 µM	2.46 + 12.17 µM	4.92 + 24.34 µM	9.84 + 48.68 µM

**Table 2:** Six selected doses for the combination experiments of **AM12** + quercetin.

Compound	0.062 × IC_50_	0.25 × IC_50_	0.5 × IC_50_	IC_50_	2 × IC_50_	4 × IC_50_
Quercetin	0.19 µM	0.74 µM	1.48 µM	2.96 µM	5.92 µM	11.84 µM
**AM12**	0.76 µM	3.04 µM	6.08 µM	12.17 µM	24.34 µM	48.68 µM
Quercetin + **AM12**	0.19 + 0.76 µM	0.74 + 3.04 µM	1.48 + 6.08 µM	2.96 + 12.17 µM	5.92 + 24.34 µM	11.84 + 48.68 µM

**Table 3:** Calculated values for the combination index of **AM12** + curcumin and **AM12** + quercetin for the reduction in the chymotrypsin-like activity of the human erythrocyte 20S proteasome.

Inhibited Fraction (fa)	% *β*5 Subunit Inhibition	Combination	CI	Effect	Combination	CI	Effect
0.50	50%	**AM12** + Curcumin	0.71	Synergism	**AM12** + Quercetin	1.04	Additive effect
0.60	60%	**AM12** + Curcumin	0.60	Synergism	**AM12** + Quercetin	1.03	Additive effect
0.70	70%	**AM12** + Curcumin	0.50	Synergism	**AM12** + Quercetin	1.04	Additive effect
0.80	80%	**AM12** + Curcumin	0.43	Synergism	**AM12** + Quercetin	1.11	Slight antagonism
0.90	90%	**AM12** + Curcumin	0.38	Synergism	**AM12** + Quercetin	1.31	Moderate antagonism

## Data Availability

The original contributions presented in the study are included in the article, further inquiries can be directed to the corresponding author/s.
